# Value of a web-based pediatric drug information system to prevent serious adverse drug reactions in child and adolescent psychiatry

**DOI:** 10.1007/s00702-022-02563-9

**Published:** 2022-11-02

**Authors:** Stefanie Fekete, Christine Kulpok, Regina Taurines, Karin Egberts, Julia Geissler, Manfred Gerlach, Dorothée Malonga Makosi, Jochem König, Michael S. Urschitz, Irmgard Toni, Antje Neubert, Marcel Romanos

**Affiliations:** 1 Department of Child and Adolescent Psychiatry, Psychosomatics and Psychotherapy, Center of Mental Health, University Hospital of Wuerzburg, Margarete-Höppel-Platz 1, 97080 Würzburg, Germany; 2 Department of Paediatrics and Adolescent Medicine, Universitätsklinikum Erlangen, Loschgestraße 15, 91054 Erlangen, Germany; 3 Division of Paediatric Epidemiology, Institute of Medical Biostatistics, Epidemiology, and Informatics, University Medical Center of the Johannes Gutenberg University, Mainz, Germany

**Keywords:** Adverse effects, Pharmacovigilance, Drug safety, Psychotropic drugs, Mental health

## Abstract

Psychotropic drugs are frequently prescribed ‘off-label’ to children and adolescents and carry the risk of serious adverse drug reactions (sADR). We examined the frequency of sADRs of psychotropic drugs in pediatric inpatients and explored their potential preventability through following the recommendations of a web-based pediatric drug information system (PDIS). The potential socio-economic impacts of using this online system is also addressed. Routine clinical data from all inpatients treated in a child and adolescent psychiatry department between January 2017 and December 2018 were retrospectively examined for the occurrence of sADRs as defined by the European Medicines Agency. The preventability of the sADRs was assessed based on the information of the PDIS. Furthermore, the expected prolongation of the hospital stay due to sADRs was calculated as well as the associated treatment costs. The study was supported by the Innovation Fund of the Joint Federal Committee, grant number 01NVF16021. In total, 1036 patients were screened of whom 658 (63.5%) received psychopharmacological treatment. In 53 (8.1%) of these patients 54 sADRs were documented, of which 37 sADRs were identified as potentially preventable through PDIS. Mitigating sADR through PDIS would likely have prevented prolonged hospital stays and conferred considerable savings for health insurance companies. PDIS provides systematic and evidence-based information about pediatric psychopharmacotherapy and helps to prevent prescribing errors. Therefore, PDIS is a useful tool to increase drug therapy safety in child and adolescent psychiatry. Further prospective studies are needed to confirm the results.

**Supplementary Information:**

The online version contains supplementary material available at 10.1007/s00702-022-02563-9.

## Introduction

Children and adolescents with mental disorders are disadvantaged in terms of drug safety and efficacy when therapy with psychotropic drugs is indicated. Very few psychotropic drugs are approved for specific indications or certain age groups (Kolch and Plener 2016). Therefore, in child and adolescent psychiatry, psychopharmacological treatment is often provided under ‘off-label’ conditions (Schroder et al. 2017). In these cases, the choice and dosage of psychotropic drugs are often solely based on empirical experience or guideline recommendations that refer to controlled studies with small sample sizes or open clinical trials (Zhou et al. 2020; Putignano et al. 2019).

Therefore, children and adolescents with mental disorders are at higher or unknown risk for serious adverse drug reactions (sADRs) (Gerlach and Warnke 2016). sADRs in children and adolescents treated with psychotropic drugs have been reported in clinical trials (Nor Aripin et al. 2012) and everyday clinical practice (Aagaard et al. 2010; Egberts et al. 2022) and can have far-reaching consequences. There is an urgent need for a strict risk–benefit assessment and careful patient and drug monitoring (Egberts et al. 2015).

A new approach to move away from practice-based, empirical to systematic and evidence-based pediatric pharmacotherapy is to provide structured and evidence-based information on the use of medications in children and adolescents, such as the currently developed and established web-based pediatric drug information system (PDIS) “kinderformularium.de” (Neubert and Rascher 2018; Zahn et al. 2021). PDIS is a transparent and evidence-based prescribing aid for clinicians including both information on licensed and off-label prescribed (psychotropic) drugs in children and adolescents (Zahn et al. 2021; Neubert und Rascher 2018). It contains dosing recommendations as well as pharmacological and pharmaceutical information for drugs, including psychotropic drugs, used in children and adolescents in Germany. PDIS aims to prevent prescribing errors and thus sADRs.

The objective of the present study was to explore the frequency of sADR in inpatients in a child and adolescent psychiatry department and the potential preventability of these sADRs through PDIS. The prolongation of the hospital stay due to the sADR was estimated, and the potential cost savings, if the prolonged stay had been prevented, were calculated to understand the potential impact of this resource. The study was conducted as a subproject of the KiDSafe project, funded by the Innovation Committee of the Federal Joint Committee under grant number 01NVF16021.

## Method

### Study design

This monocentric, retrospective clinical cohort study was conducted in accordance with the Declaration of Helsinki (World Medical Association 2013). Due to the retrospective use of anonymized health care data, no written informed parental consent was obtained. The ethics committee of the University of Wuerzburg evaluated the project positively (245/18). A positive opinion by the local data protection officer was obtained prior to data extraction.

### Study population and setting

The study was conducted at a university hospital of child and adolescent psychiatry. The department has a child psychiatric intensive care unit with 14 treatment places, 2 therapy wards with 16 treatment places each, and a specialized clinic for children and adolescents with multiple disabilities and mental disorders with 15 treatment places.

**Fig. 1:**
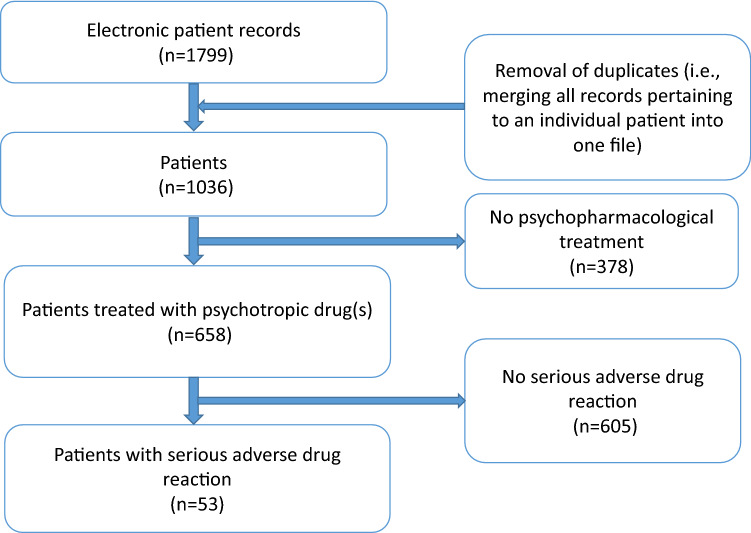
Flowchart for the identification of the study population

All electronic patient records of the child and adolescent psychiatric patients discharged from inpatient treatment between January 1, 2017, and December 31, 2018, were screened. Patient records were merged for patients admitted more than once during the study period. All patients receiving any kind of psychotropic drugs (antipsychotics, antidepressants, ADHD medication, sedatives, antiepileptics) were selected for further investigation. This population included both patients who were already receiving at least one psychotropic drug on admission, as well as patients who had a psychotropic drug (re)started, switched to another, or had their dose changed during their inpatient stay (see Fig. 1).

### Sample size calculation

The reference study for estimating the frequency of sADR was the TDM-VIGIL study with 8.3% sADR (Egberts et al. 2022). An estimated sample size of 1600 electronic medical records of hospitalized patients was provided, with approximately half of the patients having at least 1 psychotropic medication documented during the inpatient stay. Based on this value, a total number of 60 sADRs was expected.

### Data collection

The electronic patient records of all patients admitted during the study period were manually reviewed by assistant researchers under the supervision of senior physicians. Patient characteristics (age, sex), (main) psychiatric diagnoses, and all psychiatric drugs administered were recorded. Regarding the psychopharmacological treatment, it was not recorded whether the drugs were prescribed in parallel or consecutively in the whole sample. In the patients with sADR, however, drug regimens were examined and documented in detail. Age at the time of the first admission was used for analysis.

In addition, the electronic patient files of the psychopharmacologically treated patients were assessed regarding serious adverse events (SAE) (according to the definition in human drug trials [https://www.fda.gov/safety/medwatch/howtoreport/ucm053087.htm]) using a checklist based on the Pediatric Adverse Event Rating Scale (PAERS) (March et al. 2007) (see Supplementary Table 1). Discharge letters, consultation reports, inpatient documentation (medical and nursing notes), and clinical findings (vital signs, laboratory parameters, ECG, EEG) were reviewed for all patients receiving psychotropic drug treatment to identify suspected SAE with an at least possible causal relationship to a drug (= serious adverse drug reaction, sADR).

A “possible” causal relationship, defined according to the EMA (European Medicines Agency 2017) and World Health Organization (WHO) (Uppsala Monitoring Center of the WHO 2018), is a temporal relationship between the psychotropic drug and the adverse events without ruling out that an underlying disease or another medication may be responsible for the event. According to EMA (European Medicines Agency 2017), a sADR is characterized as follows: life-threatening; requiring inpatient admission or the prolongation of an inpatient stay; leading to permanent or significant damage, disability, or congenital malformation; or leading to the death of the patient.

As described below, three guiding questions on indication, dosage, and interactions were defined to assess sADR preventability through PDIS. The preventability assessment was evaluated separately by two experienced senior physicians specializing in child and adolescent psychiatry (SF, CK), discussed jointly, and finally evaluated through a consensus process.

### Treatment prolongations and excess costs due to sADR

As a measure for the ‘prolongation of the hospital stay’ due to a sADR, the duration (in days) until the symptoms of the sADR subsided after stopping the medication or reducing the dose was determined from the patient documentation.

To estimate the economic impact of preventing sADR through PDIS, we calculated the ‘prolongation of the hospital stay’ of those patients in whom a sADR occurred and were assessed as preventable through PDIS.

Saved costs were calculated by multiplying those prolongation days and the average daily rate per patient based on the current flat-rate remuneration system for psychiatry and psychosomatics (PEPP) system (therapy wards: approximately 300€/day; specialized clinic for children and adolescents with multiple disabilities and mental disorders: approximately a minimum of 400€/day; intensive care unit: approximately 600€/day) (PEPP-Entgelttarif 2017).

### Web-based pediatric drug information system (PDIS)

The PDIS aims to improve safety of drug therapy of children and adolescents. More information on the background and benefits can be found at www.kinderformularium.de (Kinderformularium.de 2021) and was recently published by Zahn and colleagues (Zahn et al. 2021).

The potential of preventability of a sADR using PDIS was defined by the following three question: Is the sADR based on (i) an incorrect indication(s) for the substance, (ii) a dosing error, or (iii) an interaction with another drug? According to the definition, an erroneous indication was present if the psychotropic drug was not named in PDIS as a possible indication for the disorder, i.e. if there was no or insufficient evidence. Dosages were classified as incorrect if they deviated from the recommendations in PDIS. In addition, the warnings stored in PDIS were checked for possible interactions with the drugs prescribed at the time of the sADR. As soon as one of these aspects was affirmed, the sADR was classified as potentially preventable.

### Statistical analysis

Statistical analysis was performed using SPSS software, version 26. Descriptive results are reported with mean, ± standard deviation (SD), and range. Statistical significance was defined as two-sided *p* < 0.05. Group difference with respect to length of stay between patients with and without a sADR was assessed with the Mann–Whitney *U* test. The consensus procedure for assessing the preventability of the sADR was presented by the coefficient of agreement using Cohen's kappa coefficient (Cohen 1960). The data set generated and analyzed during the current study is available from the corresponding author on reasonable request.

## Results

### Patient characteristics

A total of 1036 inpatients (57% female, mean age 14.2 ± 2.6 years) with 1799 admissions within the study period (2017–2018) were included in the analysis. The demographic data of the study population can be found in Table 1.

**Table 1:** Patient characteristics of the total study population (*n* = 1036)

	*n*
Setting	
Therapy wards, *n*	328
Intensive Care Unit, *n*	397
Specialized clinic for children and adolescents with multiple disabilities and mental disorders, *n*	112
Therapy wards + Intensive Care Unit, *n*	188
Specialized clinic for children and adolescents with multiple disabilities and mental disorders + Intensive Care Unit, *n*	9
Therapy wards + Specialized clinic for children and adolescents with multiple disabilities and mental disorders, *n*	1
Therapy wards + Specialized clinic for children and adolescents with multiple disabilities and mental disorders + Intensive Care Unit, *n*	1
Sex m/w, *n* (%)	444/592 (43/57)
Age (years) (SD, range)	14.2 (SD 2.6, 5–22)
Patients treated with psychotropic drugs, *n* (%)	658 (63.5)
Self-harming behavior, *n* (%)	331 (31.9)
Epilepsy, *n* (%)	37 (3.6)
Main diagnoses of mental disorders (ICD-10)	
Organic, including symptomatic mental disorders (F0), *n* (%)	1 (0.1)
Mental and behavioral disorders caused by psychotropic substances (F1), *n* (%)	14 (1.4)
Schizophrenia spectrum disorders (F2), *n* (%)	26 (2.5)
Affective disorders (F3), *n* (%)	340 (32.8)
Neurotic, stress, and somatoform disorders (F4), *n* (%)	198 (19.1)
Behavioral disorders with physical disorders and factors (F5), *n* (%)	48 (4.6)
Personality and behavioral disorders (F6), *n* (%)	30 (2.9)
Intelligence disorder (F7), *n* (%)	7 (0.7)
Developmental disorders (F8), *n* (%)	68 (6.6)
Behavioral and emotional disorders with onset in childhood and adolescence (F9), *n* (%)	304 (29.3)

ward x + ward y = inpatient stays took place consecutively on both wards during the survey period

More than half of the patients (60.1%) had at least one other comorbid psychiatric disorder (i.e. at least a second ICD-10 F diagnosis) in addition to the main psychiatric diagnosis. 658 patients (63.5%) received psychopharmacological treatment. Table 2 compares the demographics of psychopharmacologically treated patients with and without a sADR. Patients treated in the general child and adolescent psychiatry (therapy ward and intensive care unit), and patients with multiple disabilities and mental disorders treated in the specialized clinic were assessed separately.

**Table 2:** Comparison of demographics of psychopharmacologically treated patients with and without a serious adverse drug reaction (sADR)

	Patients treated in the therapy ward and/or ICU		CAMD treated in the specialized clinic	
	Patients with sADR	Patients without sADR	Patients with sADR	Patients without sADR
*N* (%)	30 (5.8)	513	23 (20.0)	92
Sex				
Male, *n* (%)	13 (43)	227 (44)	12 (52)	65 (71)
Female, *n* (%)	17 (57)	286 (56)	11 (48)	27 (29)
Age (SD, Range) (Jahre)	15.4 (1.6; 10–18)	14.1 (2.7; 5–22)	14,5 (3,0; 8–19)	13,2 (3,5; 7–19)
Self-harming behavior, *n* (%)	11 (36.7)	211 (41.1)	2 (8.7)	2 (2.2)
Epilepsy, *n* (%)	2 (6.7)	11 (2.1)	8 (34.8)	16 (7.4)
Main diagnoses of mental disorders (ICD-10)				
Mental and behavioral disorders caused by psychotropic substances (F1), *n* (%)	1 (3)	4 (1)	–	–
Schizophrenia spectrum disorders (F2), n (%)	9 (30)	8 (2)	3 (13)	3 (3)
Affective disorders (F3), *n* (%)	11 (37)	189 (37)	–	2 (2)
Neurotic, stress and somatoform disorders (F4), *n* (%)	4 (13)	85 (17)	1 (4)	3 (3)
Behavioral disorders with physical disorders and factors (F5), *n* (%)	2 (7)	16 (3)	–	2 (2)
Personality and behavioral disorders (F6), *n* (%)	–	21 (4)	2 (8)	1 (1)
Intelligence disorder (F7), *n* (%)	–	3 (1)	3 (13)	1 (1)
Developmental disorders (F8), *n* (%)	1 (3)	13 (3)	7 (30)	42 (46)
Behavioral and emotional disorders with onset in childhood and adolescence (F9), *n* (%)	2 (7)	174 (34)	7 (30)	37 (40)
Total treatment duration (days) (SD, range)	51.7 (51.3; 1–253)	28.8 (33.1, 0–344)	69.7 (53.7; 1–185)	65.4 (37.9; 1–189

*sADR* serious adverse drug reaction, *ICU* intensive unit care, *CAMD* children and adolescents with multiple disabilities and mental disorders

### Psychotropic drug prescriptions

A total of 1563 psychiatric drugs were prescribed to the patients included in the deeper analysis. Antipsychotics (*n* = 660, 42.2%) were prescribed most frequently, followed by antidepressants (*n* = 397, 25.4%) and drugs used to treat Attention-Deficit/Hyperactivity Disorder (ADHD) (*n* = 296, 18.9%) (see Table 3).

**Table 3:** Total number of psychotropic drug prescriptions and psychotropic drugs suspected to be associated with a serious adverse drug reaction (sADR)

Psychotropic drugs	Number of Psychotropic drug prescriptions suspected to be associated with sADR, *n* (%)	Number of psychotropic drug prescriptions, *n* (%)
Antipsychotics		
Aripiprazole	16 (27.6)	85 (12.9)
Quetiapine	7 (12.1)	74 (11.2)
Risperidone	6 (10.3)	89 (13.5)
Zuclopenthixol	6 (10.3)	35 (5.3)
Olanzapine	5 (8.6)	24 (3.6)
Melperone	4 (6.9)	68 (10.3)
Clozapine	4 (6.9)	4 (0.6)
Pipamperone	3 (5.2)	165 (25.0)
Chlorprothixen	3 (5.2)	50 (7.6)
Haloperidol	2 (3.4)	10 (1.5)
Thioridazine	1 (1.7)	2 (0.3)
Levomepromazine	1 (1.7)	37 (5.6)
Other		17 (2.6)
Sum	58	660
Antidepressants		
Fluoxetine	8 (36.4)	181 (45.6)
Sertraline	6 (27.3)	95 (23.9)
Escitalopram	3 (13.6)	37 (9.3)
Mirtazapine	2 (9.1)	41 (10.3)
Citalopram	1 (0.5)	6 (1.5)
Venlafaxine	1 (0.5)	14 (3.5)
Vortioxetine	1 (0.5)	1 (0.3)
Other		22 (5.5)
Sum	22	397
ADHD substances		
(Lis-) Dexamfetamin	2	77 (26.0)
Methylphenidate	1	162 (54.7)
Guanfacine	1	35 (11.9)
Atomoxetine	–	22 (7.4)
Sum	4	296
Antiepileptic drugs/Mood stabilizers		
Ethosuximide	2	2
Valproate	1	12
Oxcarbazepine	1	11
Lamotrigine	1	11
Other		16
Sum	5	52
Sedativa		
Lorazepam	1	99
Diazepam	–	16
Melatonin	–	13
Other	–	30
Sum	1	158
Total	90	1563

*ADHD* Attention-Deficit/Hyperactivity Disorder, *sADR* serious adverse drug reaction

Monotherapy with one psychotropic drug was documented in 258 patients (39.2%) during the inpatient stay; 400 patients (60.8%) received more than 1 psychotropic drug. However, whether the drugs were prescribed in parallel or consecutively was not recorded in the whole sample. On average, patients received 1.5 psychotropic drugs (SD 1.7; range 0–12) during their inpatient stay.

A total of 90 prescribed psychotropic drugs were associated with the occurrence of a sADR. The most common drug suspected of being causally related to a sADR was the second generation antipsychotic aripiprazole, which was the third most frequently prescribed antipsychotic in total.

At the time of sADR, an average of 1.7 neuro/psychotropic drugs were prescribed (SD 0.8; range 1–3). Combination treatment was suspected of causing sADRs in about half of the patients. Approximately more than two-thirds (68.9%) of the psychotropic drugs that were suspected of being related to sADR were prescribed off-label.

The antipsychotics quetiapine, olanzapine, chlorprothixene, melperone, thioridazine, and levomepromazine were dispensed off-label in all patient cases. Aripiprazole was dispensed off-label in *n* = 15 of *n* = 16 cases. In contrast, haloperidol, pipamperone, and clozapine were licensed in all cases (Kolch and Plener 2016). Risperidone was prescribed within the licensing status in half of its cases.

Among antidepressants, escitalopram, mirtazapine, citalopram, venlafaxine, and vortioxetine were dispensed off-label in all patient cases. Fluoxetine is approved in Germany for juvenile depression for ages over eight and was prescribed in seven of eight cases for this indication. Sertraline is approved in Germany for obsessive–compulsive disorder from the age of six and was prescribed in three patients for this indication and age group.

Medications for ADHD were prescribed off-label in two cases. The antiepileptic drugs valproic acid and lamotrigine were prescribed off-label. Ethosuximide and oxcarbazepine were both prescribed for the approved indication (epilepsy). Lorazepam was prescribed off-label.

### Serious adverse drug reactions (sADR)

At least 1 sADR occurred in 53 (8.1%) of the patients receiving psychotropic drugs. One patient who was admitted to the department twice suffered from a sADR during both stays, resulting in a total of 54 sADRs identified. The highest rate of sADRs was found in the subgroup of children and adolescents with multiple disabilities and mental disorders (*n* = 23; 20%).

The most common reason why ADRs were classified as ‘serious’ within this study was “inpatient treatment or prolongation of inpatient stay” caused by the ADR in 88.9% (*n* = 48) of incidents. In contrast, 9.3% of sADR were assessed as life-threatening. One patient with multiple disabilities and mental disorders died.

Furthermore, 83.3% (*n* = 45) of sADRs were somatic, and 16.7% were psychiatric (*n* = 9). The frequency of organ system affected and type of sADR is shown in Table 4. Cardiovascular ADRs (mainly QTc time prolongation) were most common, followed by extrapyramidal motor disorders.

**Table 4:** Type of serious adverse drug reaction (sADR)

Type of ADR	Description of sADR	*n* = 54
Cardiovascular ADR	QTc time prolongation (> 450 ms)	20
Nervous system ADR	EPS: Rigor, tremor, akinesia, gaze spasm, tongue or gullet spasm, akathisia, myoclonus, gait disturbance	8
	Epileptic seizure	2
Gastrointestinal ADR	Nausea, vomiting, abdominal pain with diarrhea	3
	Increased salivation	3
	Weight gain (> 10 kg)	4
Blood count change	Leukopenia	1
	Aplastic anemia	1
	Liver value increase	3
Psychiatric ADR	Suicidality	3
	Psychosis	3
	(Auto-)aggression	2
	Delirium	1

*ADR* adverse drug reaction

### Preventability by a web-based pediatric drug information system

According to the assessment of potential preventability using PDIS (“Was the indication, dosage, and interactions with the recommendations of the PDIS?”), the percentage of potentially preventable sADR was 68.5% (*n* = 37). The Cohen (1960) interrater reliability test for potential preventability showed very high agreement among the two raters (kappa = 0.92) related to the assessment of potential preventability of sADR by PDIS.

In the consensus process, *n* = 27 cases were found to have an “incorrect” indication for the substance, *n* = 5 cases had a dosing error with excessive doses, and *n* = 5 cases were suspected of having an adverse event due to drug interaction.

The criteria of preventability (indication errors, dosing errors, interaction ADR) also showed a very high level of agreement (kappa = 0.80).

### Treatment duration, inpatient prolongation, and preventable costs

There was a significant difference in treatment duration in the child and adolescent psychiatry (therapy ward and intensive care unit) between patients with sADR compared to patients without sADR (51.7 ± 51.3 days vs. 28.8 ± 33.1 days, *p* < 0.001). There was no difference in treatment duration in the specialized clinic for children and adolescents with multiple disabilities and mental disorders (69.7 ± 53.7 days vs. 65.4 ± 37.9 days, *p* = 0.656).

The estimated number of days of inpatient prolongation was done on a case-by-case basis. In *n* = 33 patients, the sADR led to an extension of inpatient stay totaling 706 days. Of these, 620 days were caused by a sADR (*n* = 22) that would have been preventable through the use of the PDIS and were, therefore, included in the calculation of cost savings. Overall, preventing these sADRs could have saved approximately €240,000 in 2 years.

## Discussion

In this retrospective study, the frequency of sADRs associated with psychotropic drug treatment in children and adolescents with mental disorders was examined in an academic inpatient setting. It assessed the preventability of sADRs through PDIS, determined the prolongation of the hospital stay due to the sADRs, and calculated the associated estimated cost to the health care system.

### Rate, type, and risk factors of sADRs

The rate of psychopharmacologically treated patients with suspected sADRs was 8.1% in the overall sample. Results from the recently completed prospective multicenter TDM-Vigil study carried out in German, Swiss, and Austrian centers (Egberts et al. 2022) showed a rate of 8.3% patients with sADRs during the entire course of the study and are thus comparable to the result of our study. In a meta-analysis of pediatric hospitalized patients (Impicciatore et al. 2001), a mean incidence of sADR of 9.5% was determined, of which 38–45% were classified as serious or life-threatening. The sADR incidence in our work is thus higher than in general pediatric patients and underlines the urgent need for intensive therapy surveillance in child and adolescent psychiatry. The highest rate of sADRs was found in the subgroup of children and adolescents with multiple disabilities and mental disorders (see Table 2), probably due to frequent comorbid somatic disorders, e.g. epilepsy. Moreover, in this group, efficacy and tolerability of medications are more difficult to assess due to the patients’ communicative impairments, which can lead to suboptimal dosing or polypharmacy.

Patients with schizophrenia (F2) and patients with intelligence disorder (F7) suffered from sADRs proportionately more often than patients with other disorders. This is probably explained by the frequent prescription of antipsychotics for these disorders (Brophy et al. 2018) and the frequent use of polypharmacy (Habetaler et al. 2015). Antipsychotics were the p

sychotropic drug class most frequently associated with sADR and were seen to be in a causal relationship with 64.4% of sADRs in this study. More broadly, antipsychotics are also frequently associated with sADRs in spontaneous reporting registries of international databases (Moore et al. 2007). Unfortunately, only a few systematic studies are available on ADRs related to antipsychotics in children and adolescents (Ben Amor 2012); there is a strong need for more systematic research in light of the increasing frequency of prescriptions (Bachmann et al. 2014). Polypharmacy was suspected to cause sADRs in half of the cases with sADR. Hilt et al. (2014) previously showed that polypharmacy with psychiatric drugs in minors increases the risk and severity of sADRs with increasing numbers of medications, especially antipsychotics, in children and adolescents (Hilt et al. 2014). However, polypharmacy is needed if more than one psychiatric disorder has to be treated pharmacologically in one patient or in cases of augmentation strategies. Therefore, Vloet and colleagues recommend using therapeutic drug monitoring (Vloet et al. 2019).

In this research, 68% of the psychotropic drugs suspected of causing a sADR were prescribed off-label. Turner et al. (1999) found pediatric medications that were not properly tested and approved were twice as likely to cause a sADR (3.9 versus 6.0%) (Turner et al. 1999). It is suspected that the high off-label use in child and adolescent psychiatry is responsible for the higher risk of ADRs (Gerlach and Warnke 2016). The prospective TDM-Vigil Study (Egberts et al. 2022) showed that about 70% of patients received psychotropic drugs off-label and that 5.6% of them had serious ADRs, while they were treated with an antidepressant or antipsychotic in off-label use. The proportion of patients with sADRs was not higher in off-label use than for treatment with approved psychotropic drugs. Schroder et al. (2017) reported a similar conclusion (Schroder et al. 2017). It should be noted that in child and adolescent psychiatry, use within the approved marketing authorization—especially for antipsychotics—is not necessarily a sign of quality, as the status is partly based on historical approvals which were granted in the past even with low level evidence, e.g., haloperidol for tic disorder from 10 years of age (see kinderformularium.de).

In the prospective TDM-Vigil study, suicide attempts were the most common type of sADR (Egberts et al. 2022). In our study, cardiac ADRs (QTc time prolongations) were the most frequently documented sADR; suicidality was documented only in three cases (5.6% of sADRs) in the titration phase of selective serotonin reuptake inhibitors (sertraline, fluoxetine, citalopram). Suicidality was documented in the patients as in causal connection with the underlying disease (depression) and was not interpreted as an ADR if suicidality had already occurred to the same extent prior to the therapy with the psychotropic drug.

### Preventability through PDIS and socio-economic benefits

When using PDIS, more than two-thirds of the sADRs were assessed as being preventable according to the three criteria defined. Most frequently, an indication for a psychotropic drug was documented in the patient record that was not listed in PDIS and thus classified as not evidence-based; e.g., for zuclopenthixol, which was frequently used in patients with multiple disabilities and “challenging behavior”, as well as the atypical antipsychotic quetiapine, which was not prescribed for the indications (psychosis, bipolar disorder), but for juvenile depression. Excessive dosages (*n* = 5), e.g., risperidone 7 mg/day, were also assessed as preventable. Moreover, interactions of two or more psychotropic drugs (*n* = 5) were indicated as a risk combination in the database, e.g., melperone and clozapine, and thus would have been preventable consulting PDIS. Inpatient treatment duration was significantly prolonged in patients with sADRs, causing high costs for the health care system.

PDIS is part of a new form of care and represents a drug information system not previously available in Germany. A particularly novel and helpful feature is that it also takes off-label applications into account and can therefore also be applied in routine care. PDIS has the potential to become a pharmacological reference for psychotropic drugs used in children and adolescents and to be integrated into routine care across the board. In other countries, such as the Netherlands, a similar database has already been implemented and has become the nationwide reference for drug dosing in children and adolescents (van der Zanden et al. 2017).

PDIS aims to ensure that recommendations, guidelines, and medications for children and adolescents are used in a more appropriate and evidence-based manner. This would facilitate the supply of medication for mentally ill children and adolescents and raise it to a level of evidence previously unknown in Germany. Although the cost savings in this study were estimated to be about a quarter of a million over 2 years, the potential preventability of life-threatening sADRs in children and adolescents is the most powerful argument for the use of PDIS.

### Limitations

The results of this study must be evaluated in the context of limitations of a retrospective study. All data were based on available routine patient records. Thus, the quality of the documentation had impact on the quality of the data. However, the department has previously participated in pharmacovigilance drug randomized controlled trials (RCTs;TDM-VIGIL) having substantially improved the standards for routine documentation on drug administration and side effects. The almost identical percentage of identified sADRs compared to TDM-VIGIL supports the assumption of a good documentation quality in our study. However, results from a monocentric study limit generalizability. The causal relationship between sADRs and psychotropic drugs was assessed using WHO criteria (Uppsala Monitoring Center of the WHO): none of the cases were classified as having a “probable” or “certain” causal relationship, because in these cases, differential diagnoses for the adverse events should have been systematically excluded, or even re-exposure to the suspected substance should have occurred. Therefore, most cases had only a “possible” causal relationship to the neuro/psychotropic drug and other causes (e.g., comorbid somatic disorders, like epilepsy, or the disorder itself, like suicidality in patients suffering from depression), could also be responsible for the occurrence of SAE. However, the assessment using WHO criteria is a standardized process, which is also used in pharmacovigilance practice in the EU. Other causes, such as severe somatic pre-existing conditions, especially in children with multiple disabilities, could therefore, also be responsible for the occurrence of the adverse events, e.g. an epileptic seizure. Neither the severity of psychiatric disorders nor the efficacy of the psychotropic drugs was assessed in this study. However, children and adolescents are treated as inpatients only if their psychiatric disorder has a serious severity. If psychopharmacological treatment is indicated, "non-treatment" will lead to severe consequences due to the psychiatric disorders (e.g. schizophrenia). This necessitates a strict risk–benefit assessment. If psychopharmacological treatment is indicated, all available pharmacovigilance measures must be exhausted (e.g. PDIS and therapeutic drug monitoring).

Regarding the prolongation of stays and the forecasted potential savings as a result of using PDIS, the results have to be interpreted with caution. The duration of inpatient stay may also have depended on many other factors not recorded in this study (such as symptom aggravation irrespective of the medication) and not adjusted for in the analysis. Additionally, the raw comparison of length of stay between patients with and without sADR is subject to immortal time bias. Therefore, the estimated number of days of prolongation was done on a case-by-case basis, and the calculation of the cost savings was also based only on rounded rough estimates.

## Conclusion

The PDIS provides systematic and evidence-based information about pediatric psychopharmacotherapy and helps to prevent prescribing errors and thus sADRs. Therefore, PDIS is a useful tool to increase drug therapy safety in the psychopharmacological treatment of children and adolescents with psychiatric disorders. Further prospective studies are needed to confirm the results.

## Supplementary Information

Below is the link to the electronic supplementary material.

Supplementary file1 (DOCX 13 kb)

## Data Availability

The datasets generated during and/or analyzed during the current study are available from the corresponding author on reasonable request.
