# Supplementary material for: Value of a web-based pediatric drug information system to prevent serious adverse drug reactions in child and adolescent psychiatry
Source: J Neural Transm (Vienna). 2022 Nov 2;130(1):53–63. doi: 10.1007/s00702-022-02563-9 (PMC9813149; doi:10.1007/s00702-022-02563-9)
Supplement: Supplementary file 1 — Supplementary file1 (DOCX 13 kb) [file 702_2022_2563_MOESM1_ESM.docx]

**Supplement Table 1:** **Adverse Drug Reaction Checklist based on the Pediatric Adverse Event Rating Scale (PAERS)** (**March et al. 2007)**

| **Organ system affected** | **Type of ADR** | **Description of ADR, examples** |
| --- | --- | --- |
| Nervous system | Sedation | Fatigue, drowsiness |
|  | Tension | Agitation, restlessness, increased urge to move, impulsivity |
|  | Extrapyramidal motor disorder (EPS) | Rigor, tremor, akinesia, gaze spasm, tongue or gullet spasm, athetosis, ballismus, chorea, dystonias, dyskinesias, fasciculations, myoclonus, myokymia, restless legs syndrome, spasm, akathisia |
|  | Epileptic seizure | Epileptic seizure, convulsive seizure |
|  | Tics | Tics (vocal, motor) |
|  | Headache | Headache, migraine |
|  | Sleep disorder | Difficulty falling asleep, difficulty sleeping through the night, hypersomnia |
| Cardiovascular system | Cardiovascular ADR | Dizziness, orthostatic Hypotension, hypertension, ECG change, QTc prolongation, AV block, chest pain, syncope, tachycardia, subjective palpitations |
| Gastrointestinal Tract | Gastrointestinal ADR | Abdominal shivering, nausea, vomiting, constipation, diarrhea |
|  | Weight change | Weight loss, weight gain |
|  | Salivation | Dry mouth, increased salivation |
| Blood formation | blood count changes | Leukopenia |
|  |  | Prolactin increase |
|  |  | Aplastic anemia |
|  |  | Liver elevation |
| Psych | Psychiatric ADR | Mood deterioration, apathy, mood changes, emotional lability, suicidal ideation, suicide attempt, suicide |
|  |  | Psychosis, hallucinations |
|  |  | Aggression (external and autoaggression) |
|  |  | Attention/concentration disorder |
| Metabolism and nutrition | Nutritional disorders | Reduced appetite, increased appetite |
|  |  | Pronounced thirst |
| Respiratory system | Respiratory ADR | Nasal airway obstruction, respiratory problems, bronchial asthma |
| Urogenital tract | Urogenital ADR | Genitourinary disorder Urinary retention, impotence, dysuria (painful), pollakisuria (frequent urination), polyuria (increased urine volume 2-4l), oliguria (<500ml), anuria (<100ml), nocturia (at night), fluor vaginalis |
| Skin | Skin disorders | Acne, skin rash |
|  |  | Bruising, bleeding |
| Other ADR | Other ADR | General malaise/sick feeling |
|  |  | Sexual dysfunction |

ADR: Adverse drug reaction
